# Supplementary material for: Exploiting evolutionary steering to induce collateral drug sensitivity in cancer
Source: Nat Commun. 2020 Apr 21;11:1923. doi: 10.1038/s41467-020-15596-z (PMC7174377; doi:10.1038/s41467-020-15596-z)
Supplement: Supplementary file 1 — Supplementary Information [file 41467_2020_15596_MOESM1_ESM.pdf]

Supplementary Information

Exploiting evolutionary steering to induce collateral drug sensitivity in cancer

Ahmet Acar[1,], Daniel Nichol[1,*], Javier Fernandez-Mateos[1], George D. Cresswell[1], Iros Barozzi[2], Sung Pil Hong[2], Nicholas Trahearn[1], Inmaculada Spiteri[1], Mark Stubbs[3], Rosemary Burke[3], Adam Stewart[4], Giulio Caravagna[1], Benjamin Werner[1], Georgios Vlachogiannis[5], Carlo C. Maley[6], Luca Magnani[2], Nicola Valeri[5,7], Udai Banerji[4,5,§], Andrea Sottoriva[1,§].*

[1] Evolutionary Genomics & Modelling Lab, Centre for Evolution and Cancer, The Institute of Cancer Research, London, UK.
[2] Department of Surgery and Cancer, Imperial College London, London, UK.
[3] CRUK Cancer Therapeutics Unit, The Institute of Cancer Research, London, UK.
[4] Clinical Pharmacology - Adaptive Therapy Group, Division of Cancer Therapeutics and Clinical Studies, The Institute of Cancer Research, London, UK.
[5] Drug Development Unit, The Institute of Cancer Research and The Royal Marsden Hospital NHS Foundation Trust, London, UK.
[6] Arizona Cancer Evolution Center, Biodesign Institute, Arizona State University, Tempe, USA.
[7] Department of Medicine, The Royal Marsden NHS Foundation Trust, London, UK.

**equal contribution (AA: wet lab work, DN: dry lab work)*

§correspondence to udai.banerji@icr.ac.uk or andrea.sottoriva@icr.ac.uk

Supplementary Figures

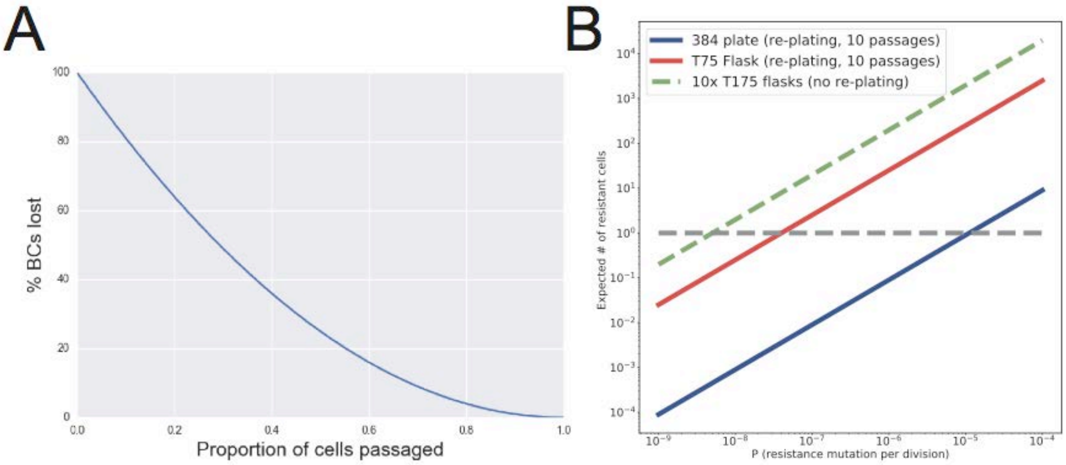

Supplementary Figure 1. Effects of stochastic evolutionary dynamics in small cell populations with re-plating. ***(A)*** *Re-plating induces population bottlenecks that lead to artificial loss of intra-tumour heterogeneity (illustrated as lineage loss assuming 2M cells re-plated with different proportions).* ***(B)*** *Expected number of de novo mutants versus mutation rate assuming 384-well plates or T175 flasks after 10 passages (1:10) versus 10x T175 flasks without re-plating.*

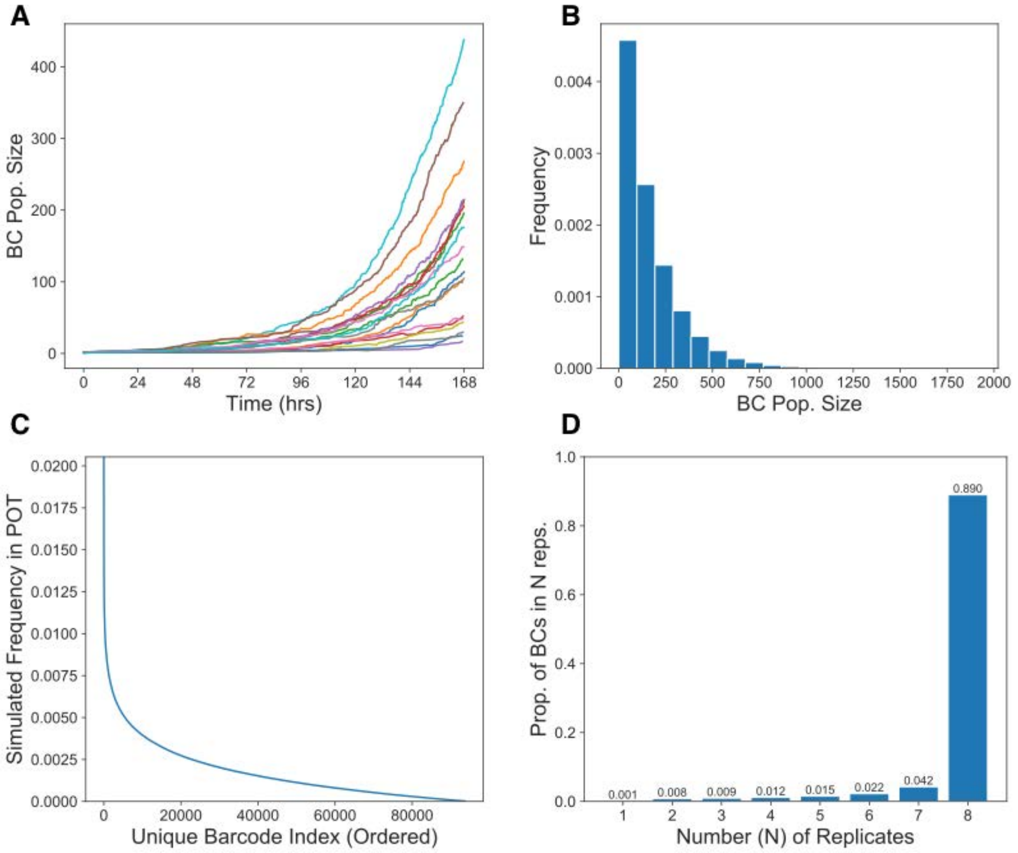

Supplementary Figure 2. Model simulation of POT expansion and splitting into replicates. **(A)** Example realisations of a stochastic model of exponential growth in a birth-death model starting from a single cell. **(B)** Histogram of final population sizes in 10,000 independent realisations of the simulation after 168hrs of growth. Simulations wherein the population is extinct are not shown. **(C)** The predicted relative frequency of each surviving barcode in the POT final population following 168hrs of growth from an initial population of 10,000 cells each with a unique barcode. **(D)** Predicted probabilities that a given barcode, corresponding to a single uniquely barcoded cell in the initial population of 10,000 cells, is present in precisely N replicates following POT expansion and 8-way splitting.

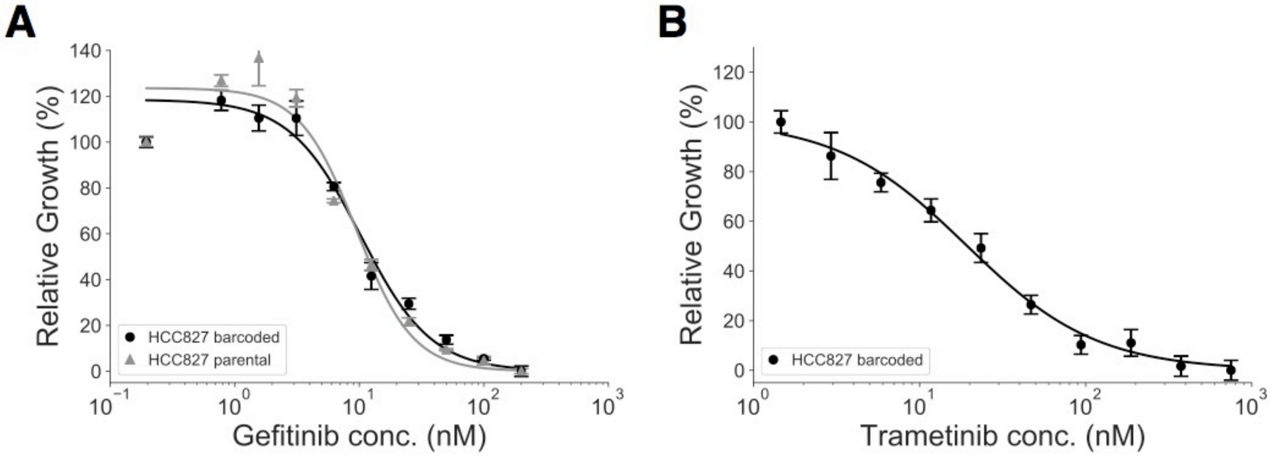

Supplementary Figure 3. Dose response curves of gefitinib and trametinib at baseline. (A) Gefitinib and **(B)** trametinib dose response curves for baseline HCC827 and corresponding barcoded lines used to estimate GI90 values for each drug. Error bars represent SEM.

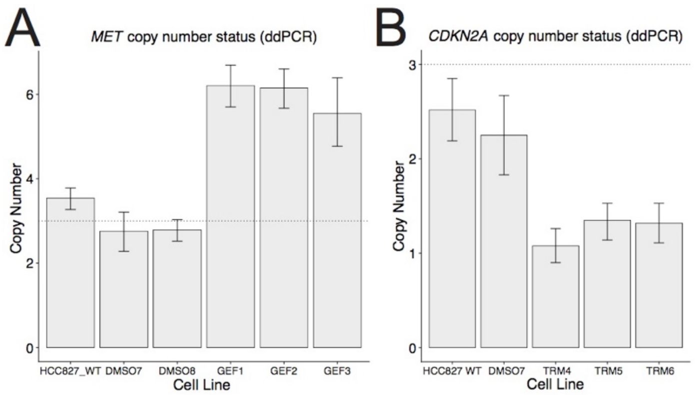

Supplementary Figure 4. *MET* amplification in gefitinib resistant lines and *CDKN2A* loss in trametinib resistant lines is confirmed by ddPCR. (A) Number of copies of *MET* go from 3 in the baseline and DMSO to 6+ in the gefitinib evolved lines. **(B)** Number of copies of *CDKN2A* go from 2/3 in the baseline to 1 in trametinib evolved lines. Bar plots represent the copy number as estimated by dividing the target locus concentration by the reference *NSUN3* locus concentration and multiplying this ratio by three as *NSUN3* is in three copies (triploid genome). Error bars represent the 95% Confidence Interval for the ratio (Total Error Model) as produced by QuantaSoft^{TM} multiplied by 3. In ddPCR it is possible to calculate confidence intervals from the results of a single well by modelling positive and negative droplets as being generated by a Poisson distribution.

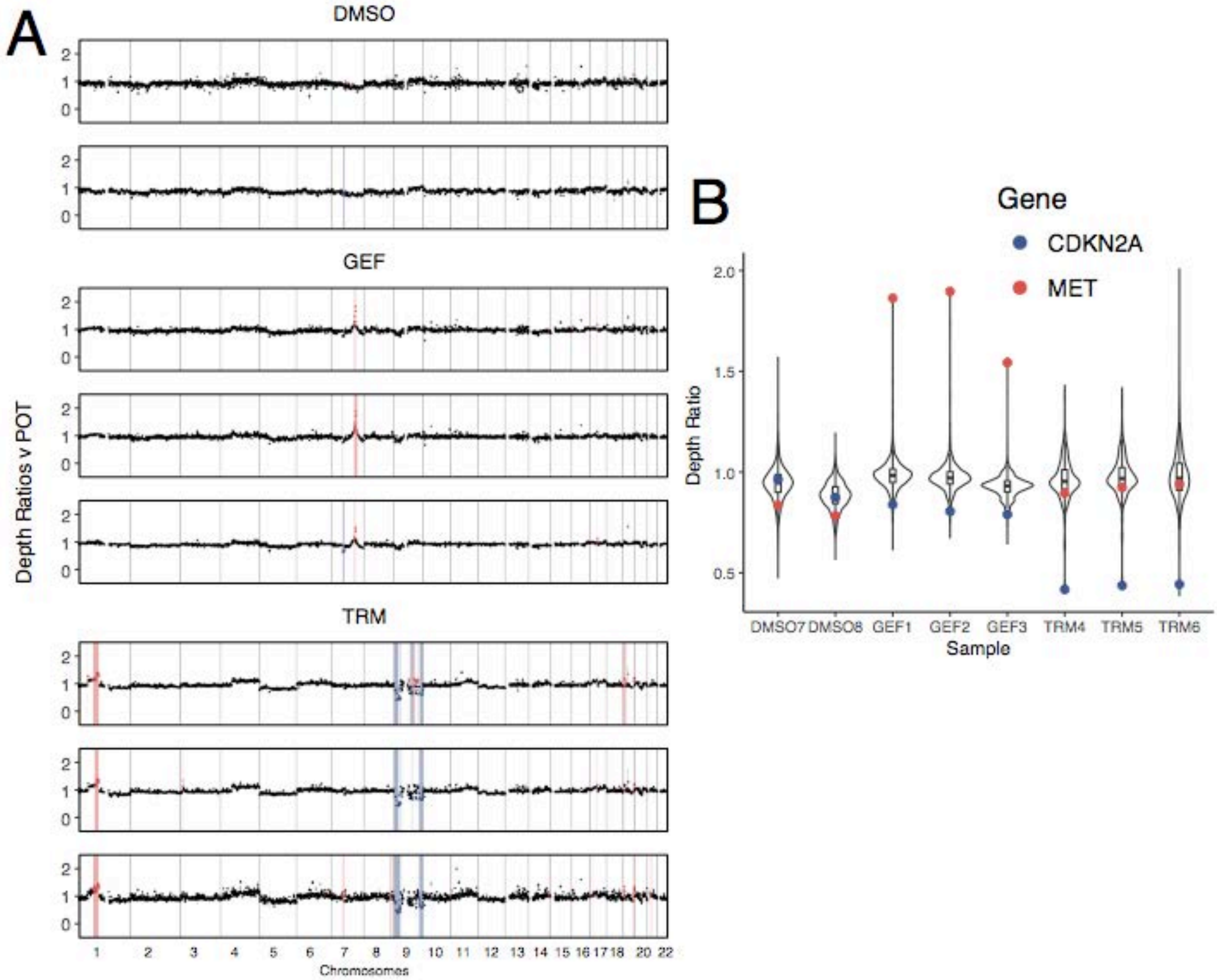

Supplementary Figure 5. Differential copy number profiles vs baseline POT. (A) Depth ratio values of samples versus POT indicate that *MET* amplification in GEF lines and +1p and -9p in TRM lines. **(B)** Depth ratio values of *MET* and *CDKN2A* compare with whole-genome depth ratios

highlight the putative driver changes in each line. Boxplots represent the median and the interquartile values.

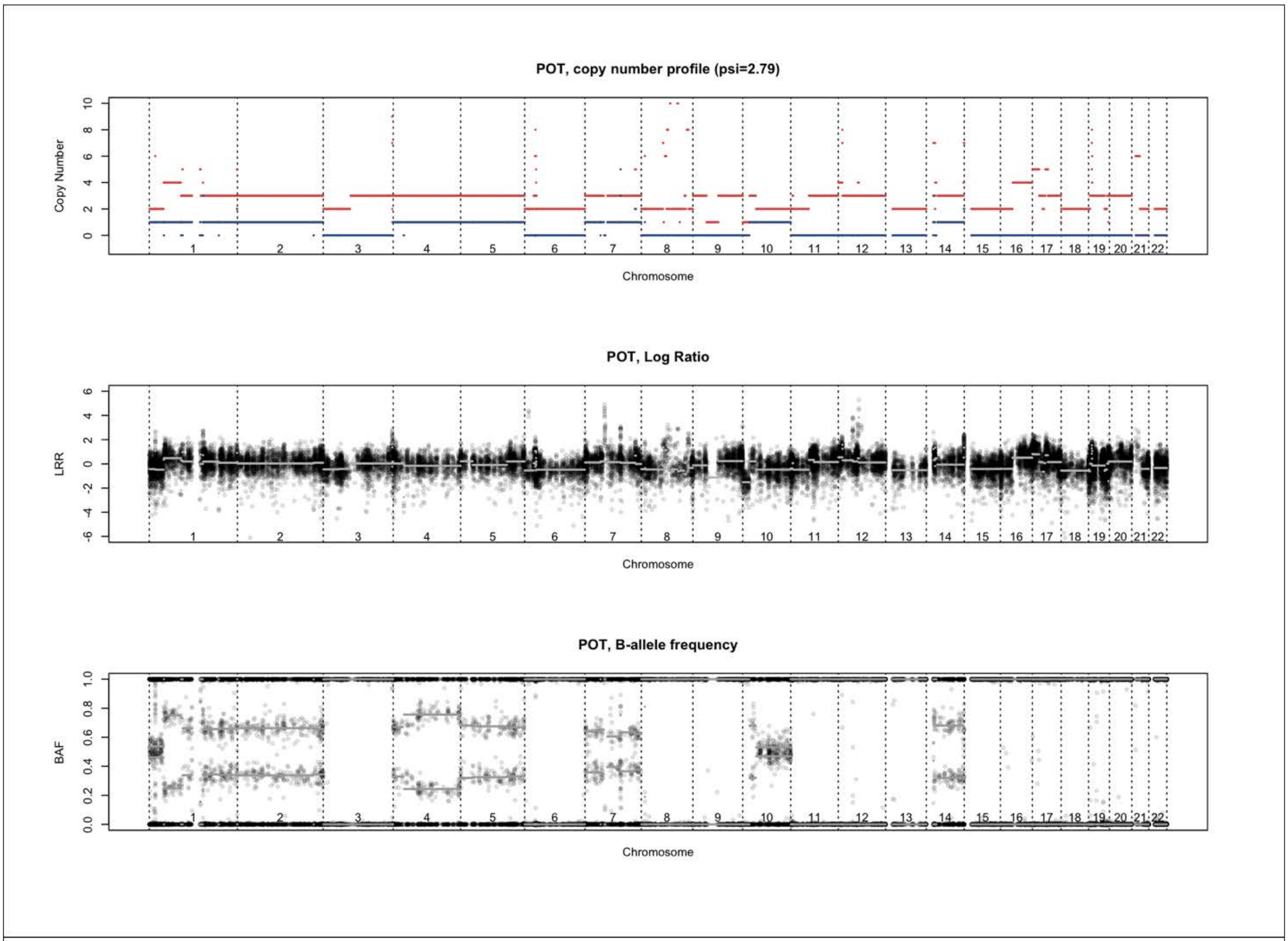

POT, copy number profile (psi=2.79)
Copy Number
Chromosome
POT, Log Ratio
LRR
Chromosome
POT, B-allele frequency
BAF
Chromosome

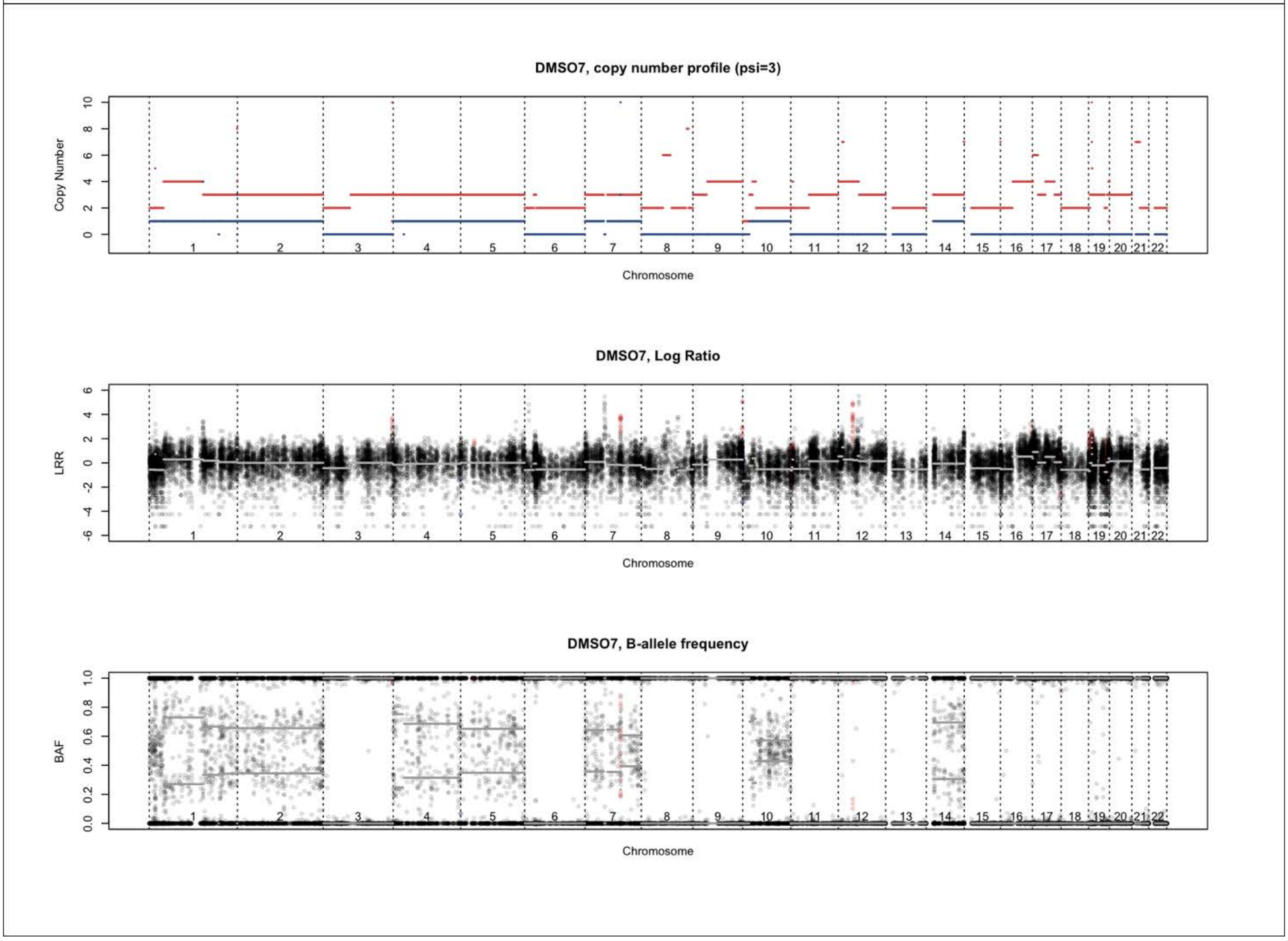

DMSO7, copy number profile (psi=3)
Copy Number
Chromosome
DMSO7, Log Ratio
LRR
Chromosome
DMSO7, B-allele frequency
BAF
Chromosome

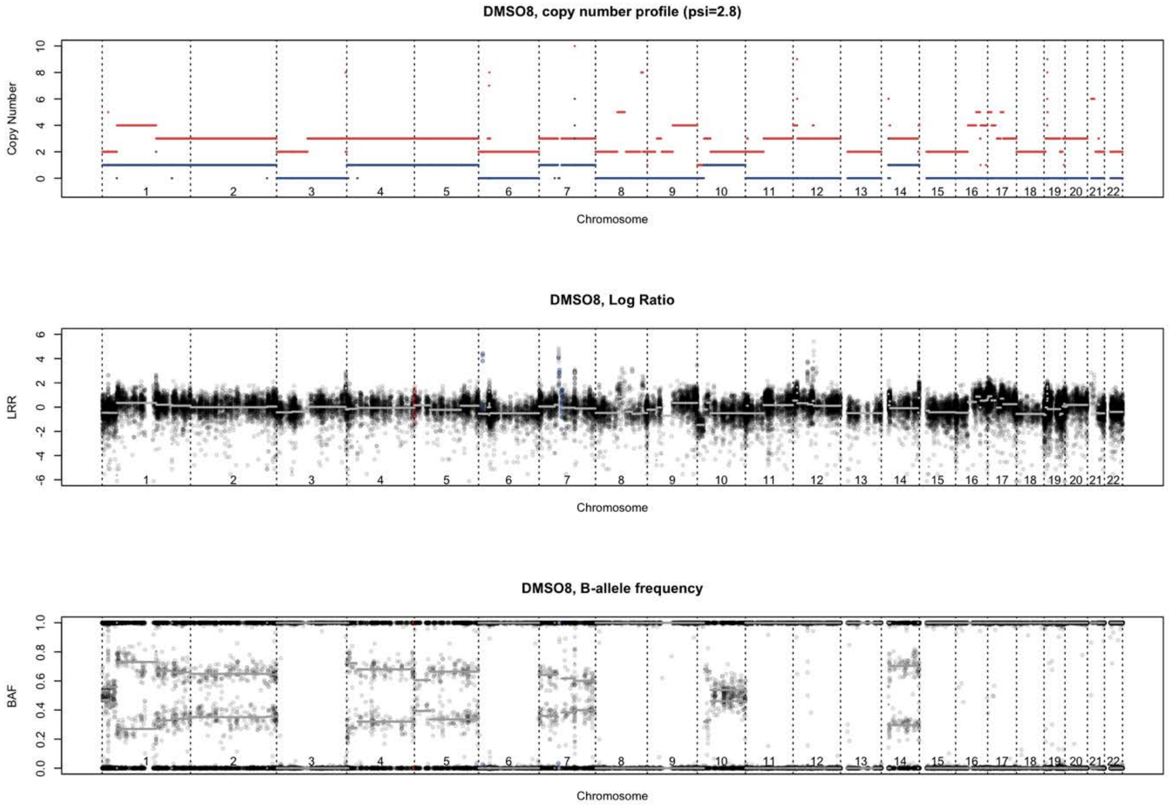
DMSO8, copy number profile (psi=2.8)
Copy Number
Chromosome
DMSO8, Log Ratio
LRR
Chromosome
DMSO8, B-allele frequency
BAF
Chromosome

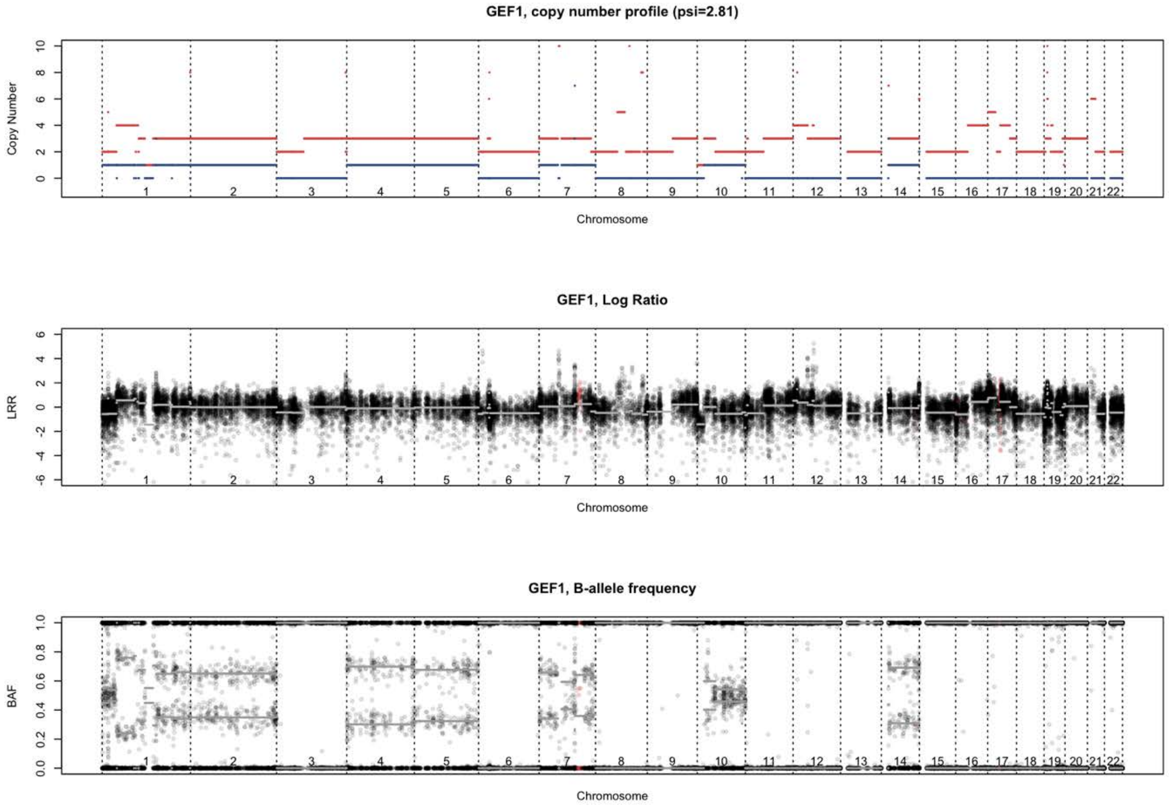
GEF1, copy number profile (psi=2.81)
Copy Number
Chromosome
GEF1, Log Ratio
LRR
Chromosome
GEF1, B-allele frequency
BAF
Chromosome

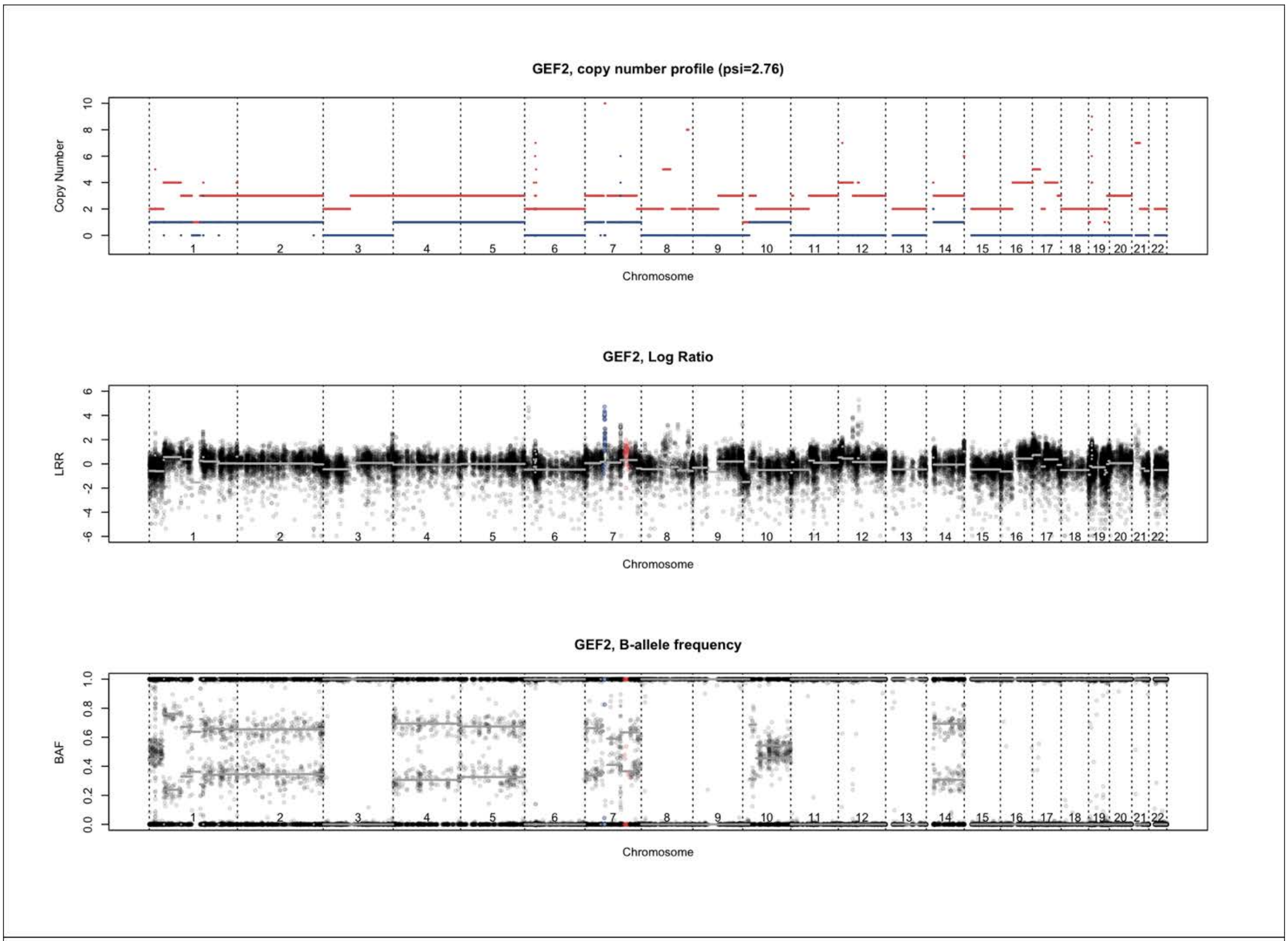
GEF2, copy number profile (psi=2.76)
Copy Number
Chromosome
GEF2, Log Ratio
LRR
Chromosome
GEF2, B-allele frequency
BAF
Chromosome

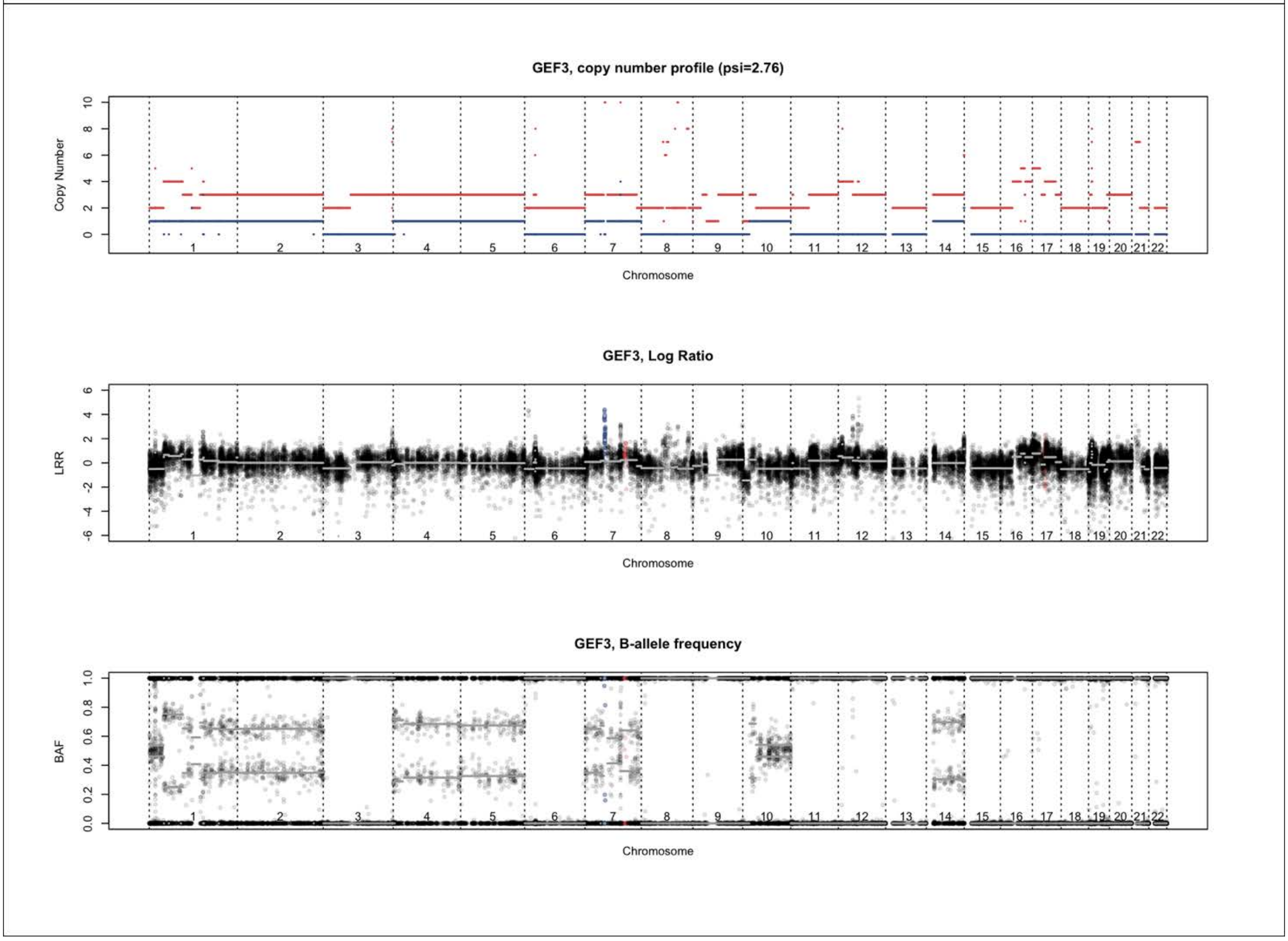
GEF3, copy number profile (psi=2.76)
Copy Number
Chromosome
GEF3, Log Ratio
LRR
Chromosome
GEF3, B-allele frequency
BAF
Chromosome

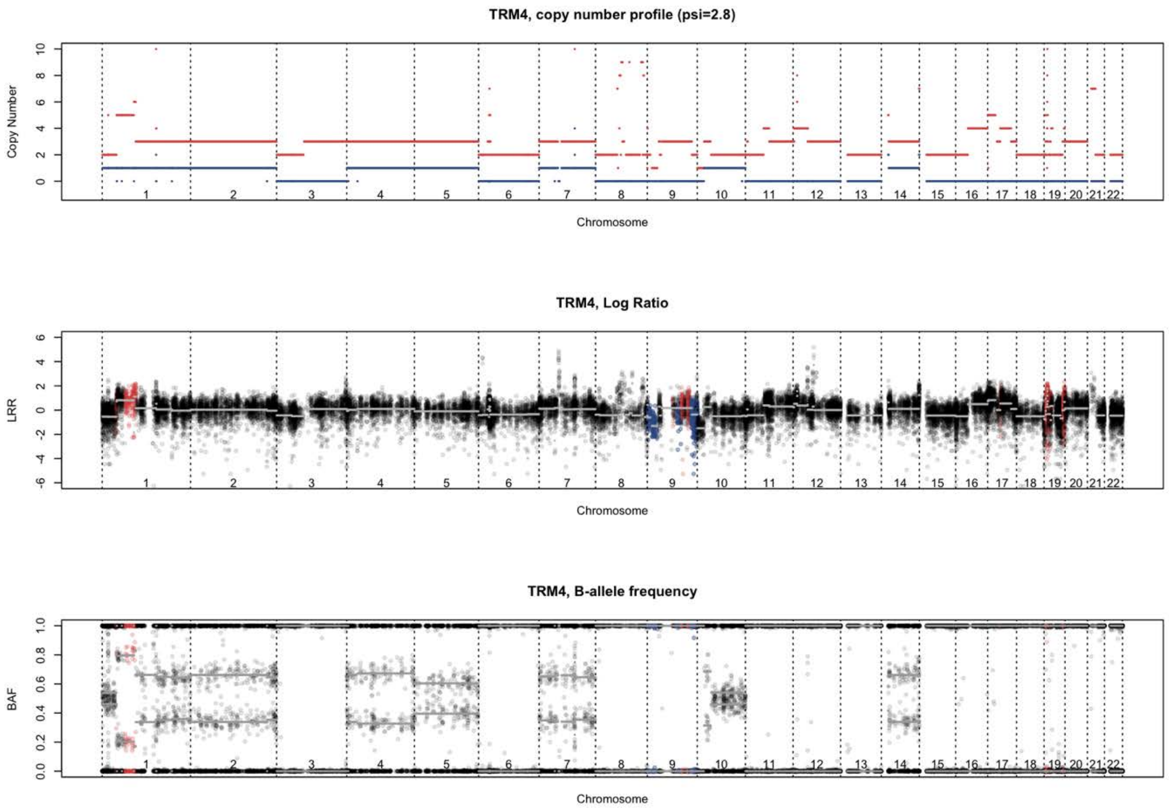
TRM4, copy number profile (psi=2.8)
Copy Number
Chromosome
TRM4, Log Ratio
LRR
Chromosome
TRM4, B-allele frequency
BAF
Chromosome

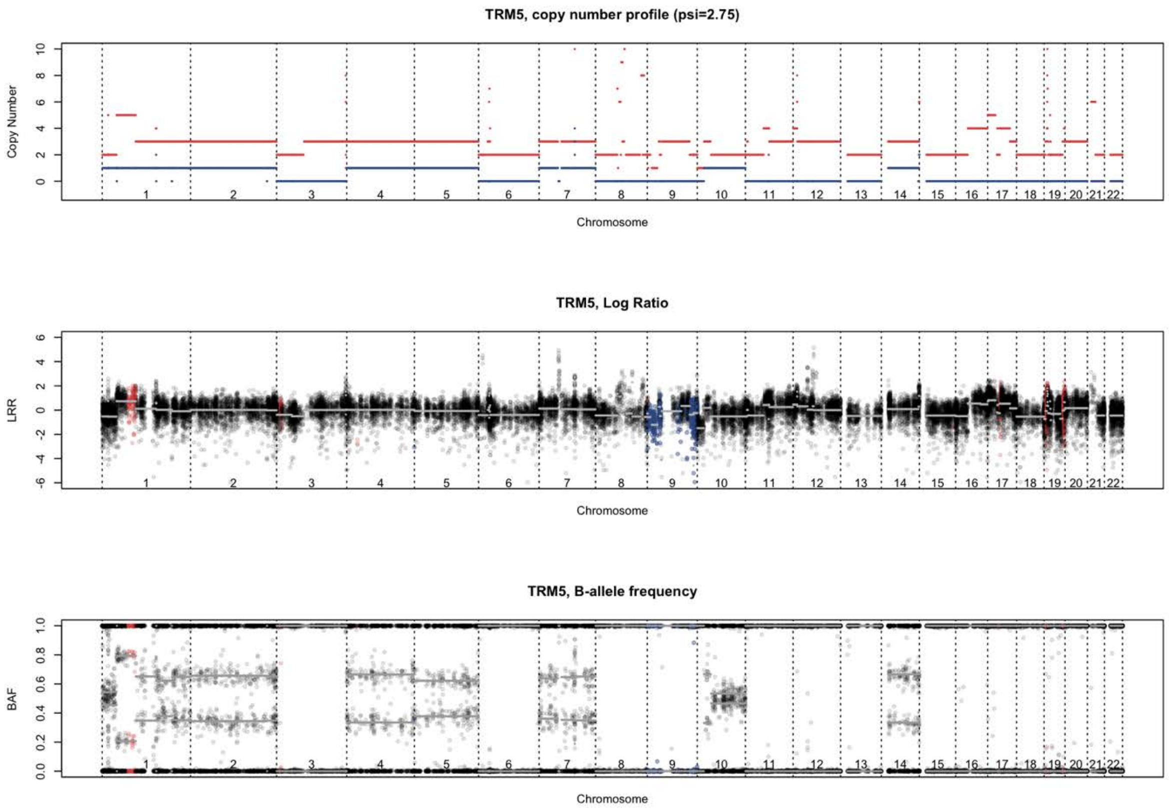
TRM5, copy number profile (psi=2.75)
Copy Number
Chromosome
TRM5, Log Ratio
LRR
Chromosome
TRM5, B-allele frequency
BAF
Chromosome

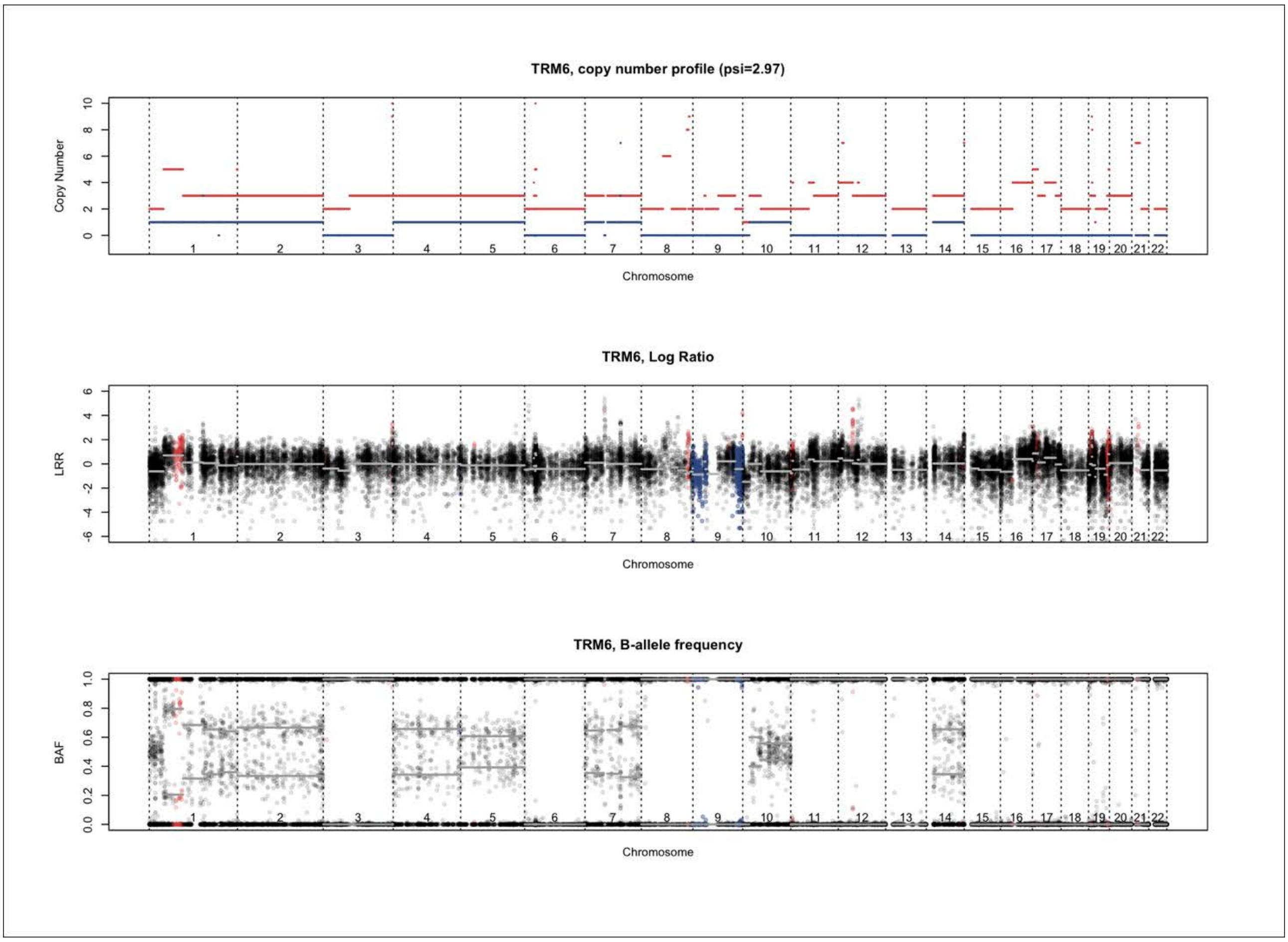

Supplementary Figure 6. Complete copy number calling per sample. Each sample absolute copy number estimates are represented. HCC827 is a triploid cell line with large proportion of the genome being in LOH. This analysis confirmed the gain of 1p and 9q in TRM lines and the amplification of MET in the GEF lines. Copy number profiles show total copy number (red) and minor allele copy number (blue). Log Ratio and B-allele frequency values are coloured according to their overlap with depth ratio segments, red if the segment is a gain and blue if the segment is a loss.

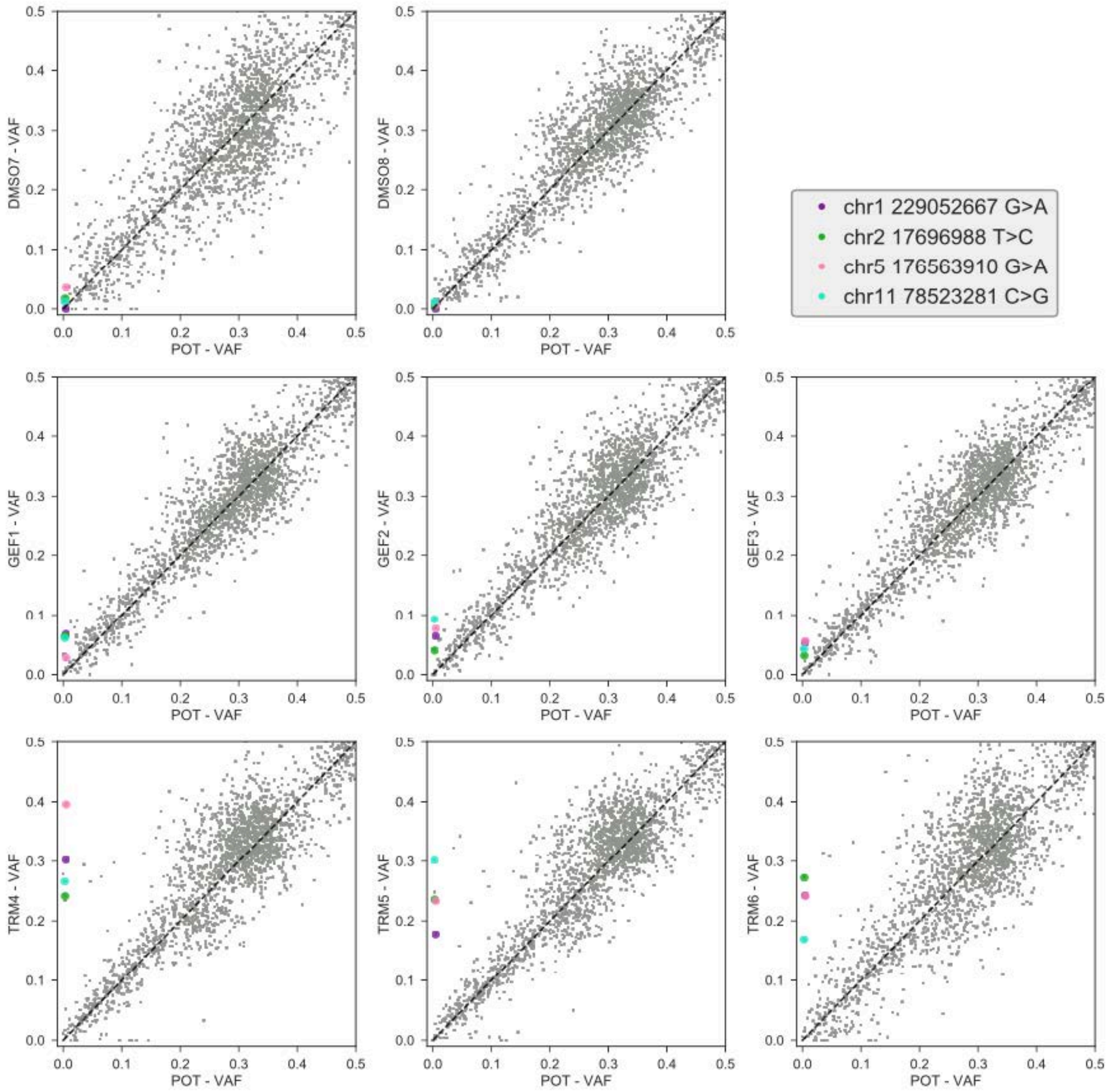

Supplementary Figure 7. SNV enrichment analysis. HCC827 is a triploid cell line, hence we expect cluster of variants at VAF=0.33 and 0.66. Subclonal mutations in a single allele that become clonal will reach clonality at VAF~0.33. A small set of mutations were enriched in all replicas of GEF and TRM, and reached almost clonality in TRM.

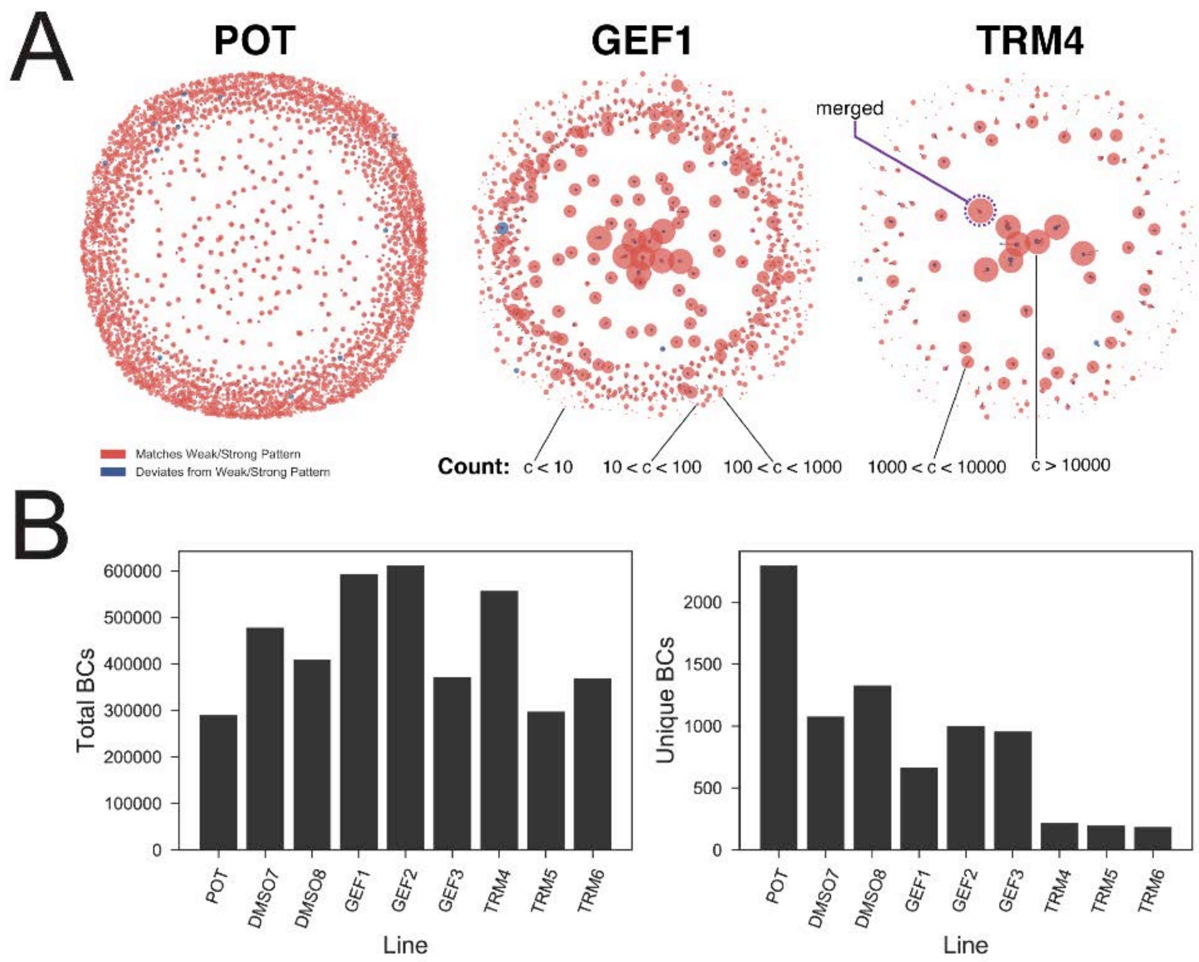

Supplementary Figure 8. Merging barcode sequences to correct for errors. (A) Example barcode sequence distance networks for POT, GEF1 and TRM4. Points represent unique barcode sequences and are connected by edges where the sequences differ by Hamming distance 2 or less. Point size is determined by the number of the unique barcode detected in the replicate. Colour indicates whether the barcode matches the weak/strong base pair pattern. Our correction algorithm merges connected components of the network (example highlighted in purple). **(B)** Total barcodes (left) and unique barcodes (right) identified in each replicate following filtering and merging.

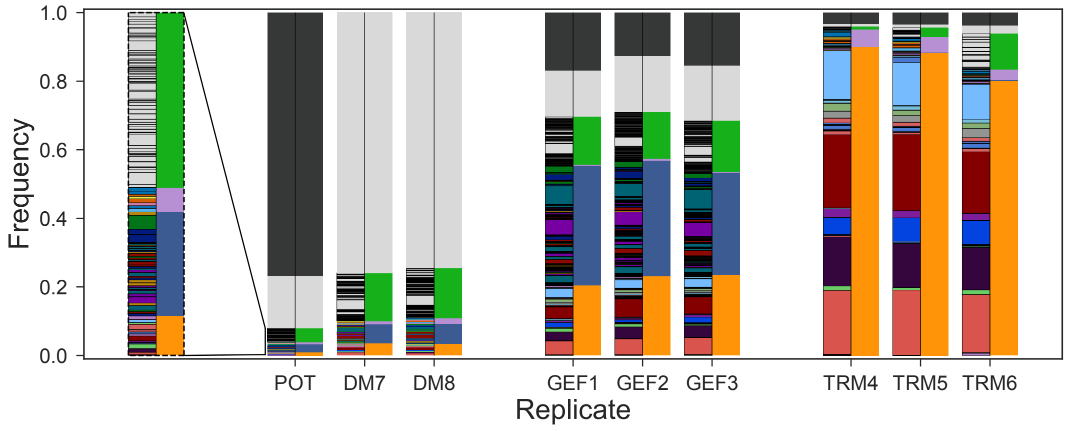

Supplementary Figure 9. Barcodes with undetermined phenotype. Barcode frequency distributions in each sample as in Figure 4B. Left hand bars show the frequency of each unique barcode. Barcode colours are ordering are identical between replicates. Right hand bars indicate the phenotypes assigned to each barcode. Here, barcodes with undetermined phenotype (those not found in DM7 or DM8) are coloured dark grey.

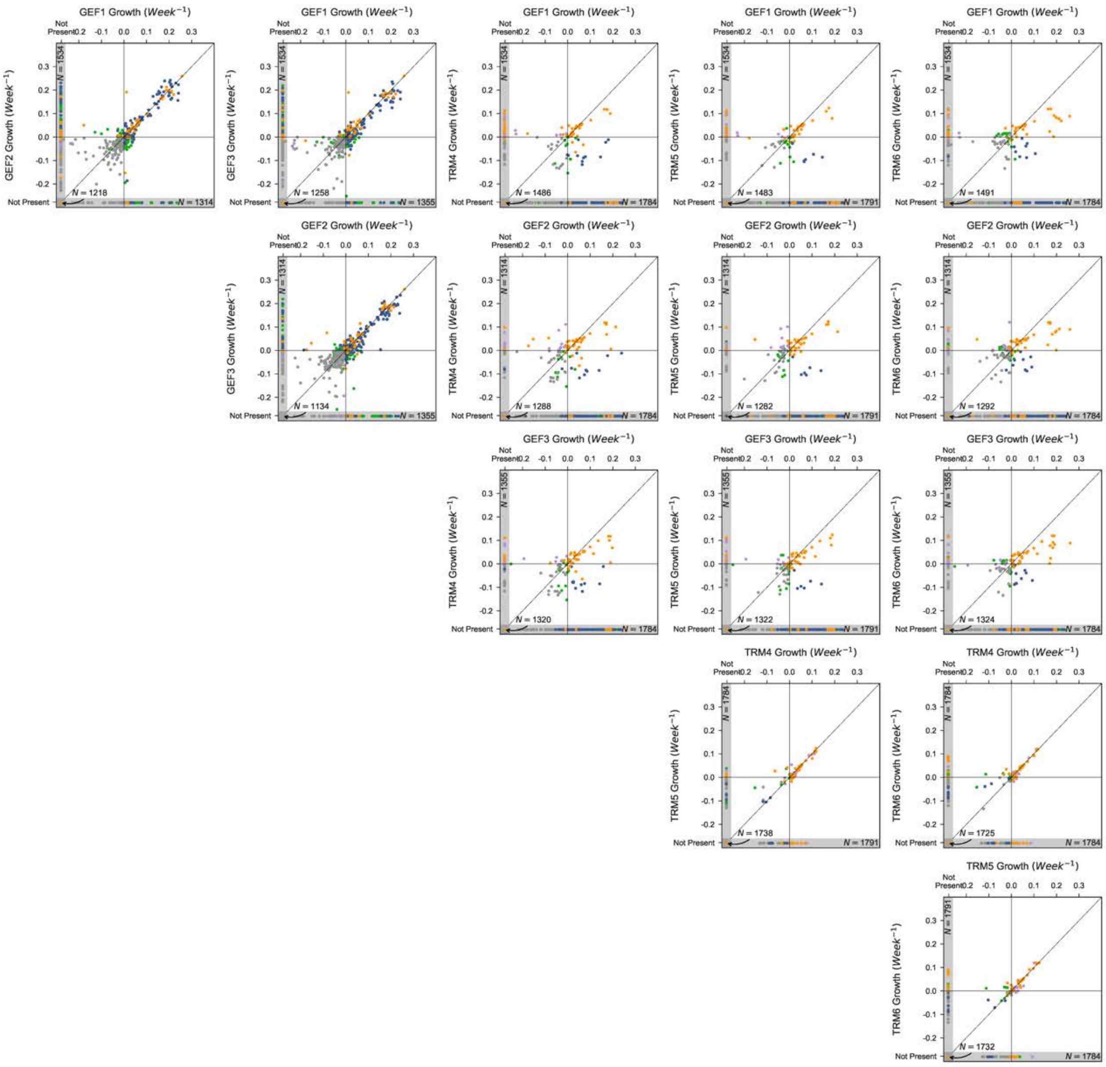

Supplementary Figure 10. Concordance in barcode growth rates between replicates. Scatter plots show the concordance in barcode growth rates between pairs of evolutionary replicates. Points are coloured according to barcode phenotype, as in Figure 4B.

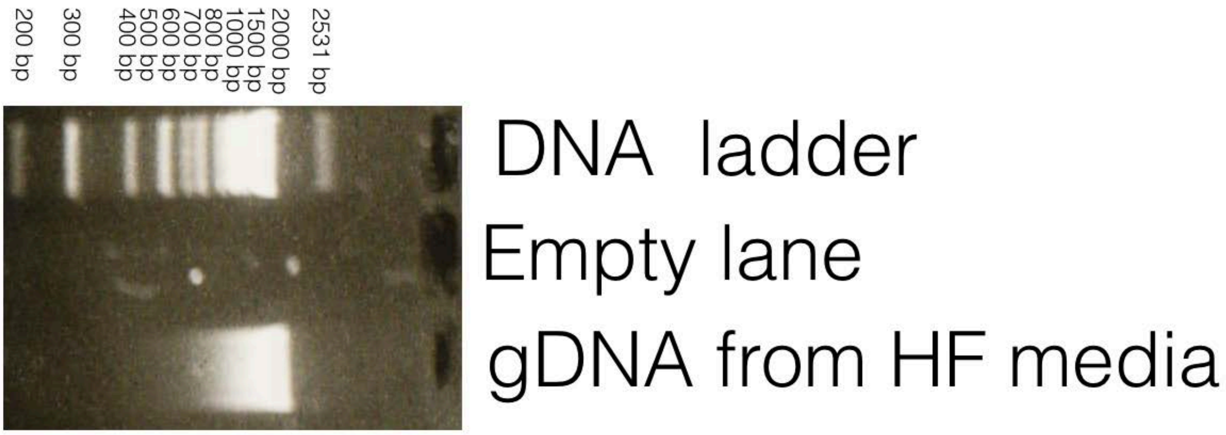

Supplementary Figure 11. DNA degradation demonstrate media floating cells are dead. DNA from floating cells in the supernatant media was degraded, consistently with DNA coming from dead cells.

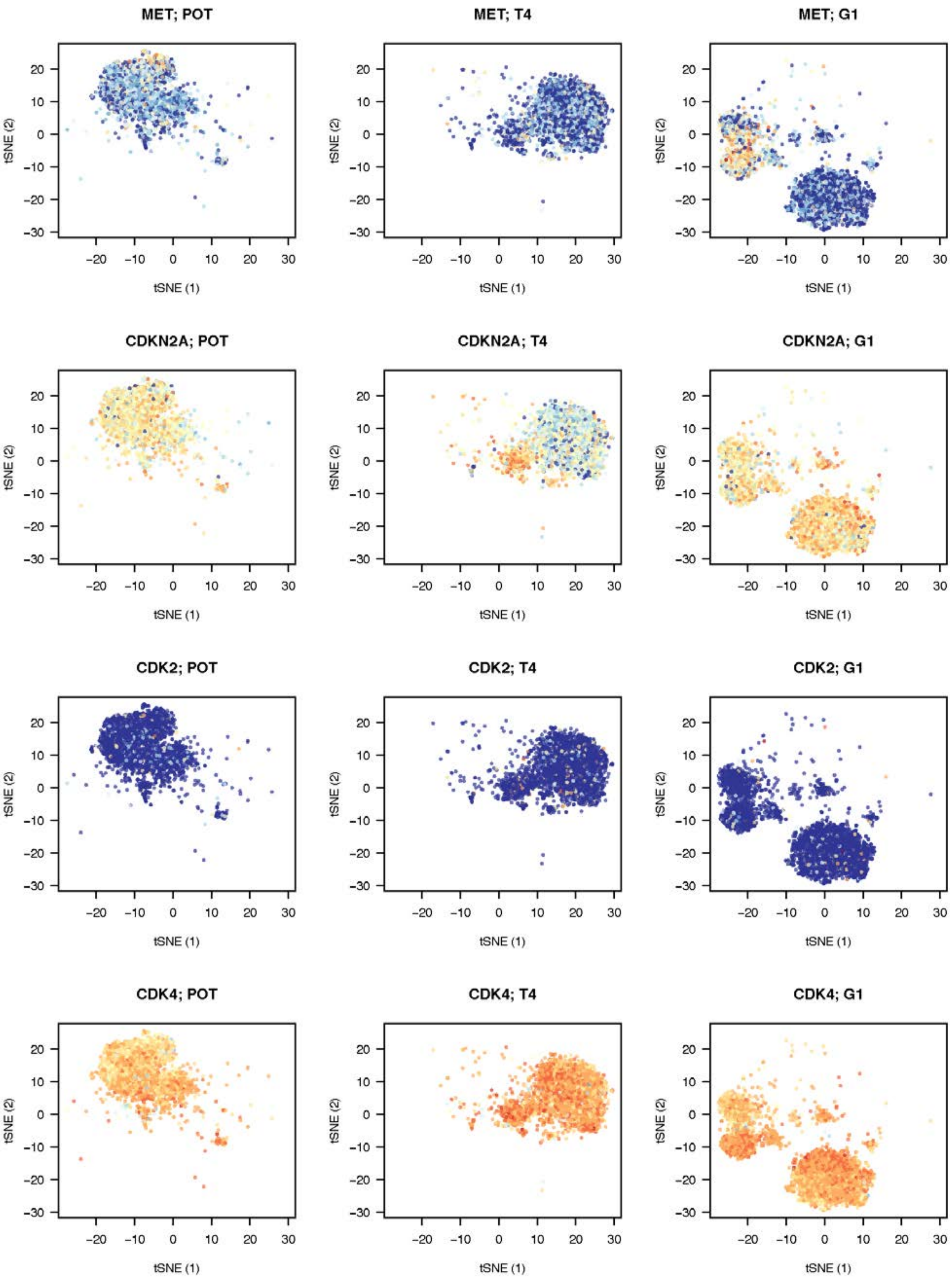
MET; POT
MET; T4
MET; G1
CDKN2A; POT
CDKN2A; T4
CDKN2A; G1
CDK2; POT
CDK2; T4
CDK2; G1
CDK4; POT
CDK4; T4
CDK4; G1
tSNE (1)
tSNE (2)
−20
−10
0
10
20
30
−30

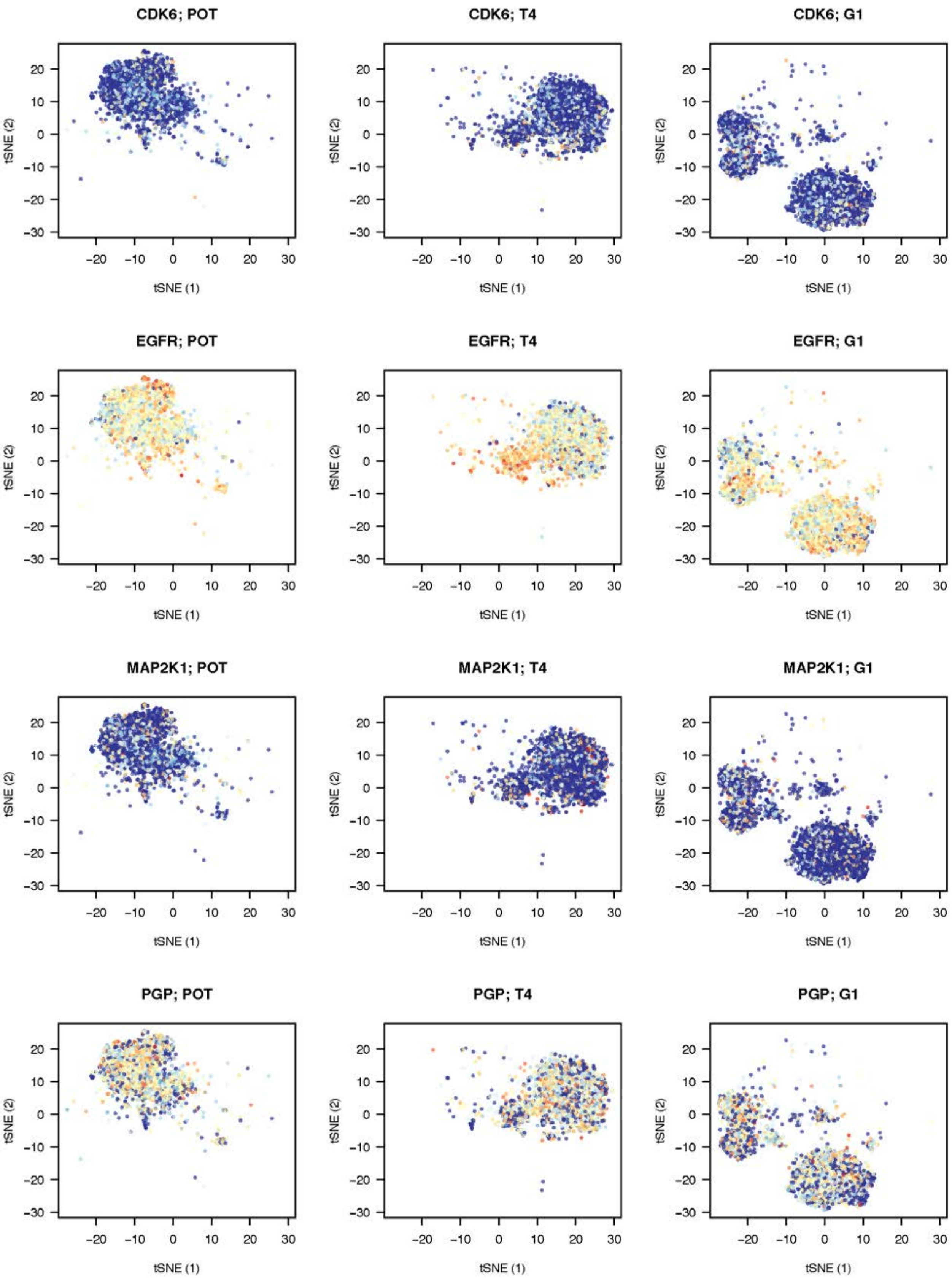
CDK6; POT
CDK6; T4
CDK6; G1
EGFR; POT
EGFR; T4
EGFR; G1
MAP2K1; POT
MAP2K1; T4
MAP2K1; G1
PGP; POT
PGP; T4
PGP; G1
tSNE (1)
tSNE (2)

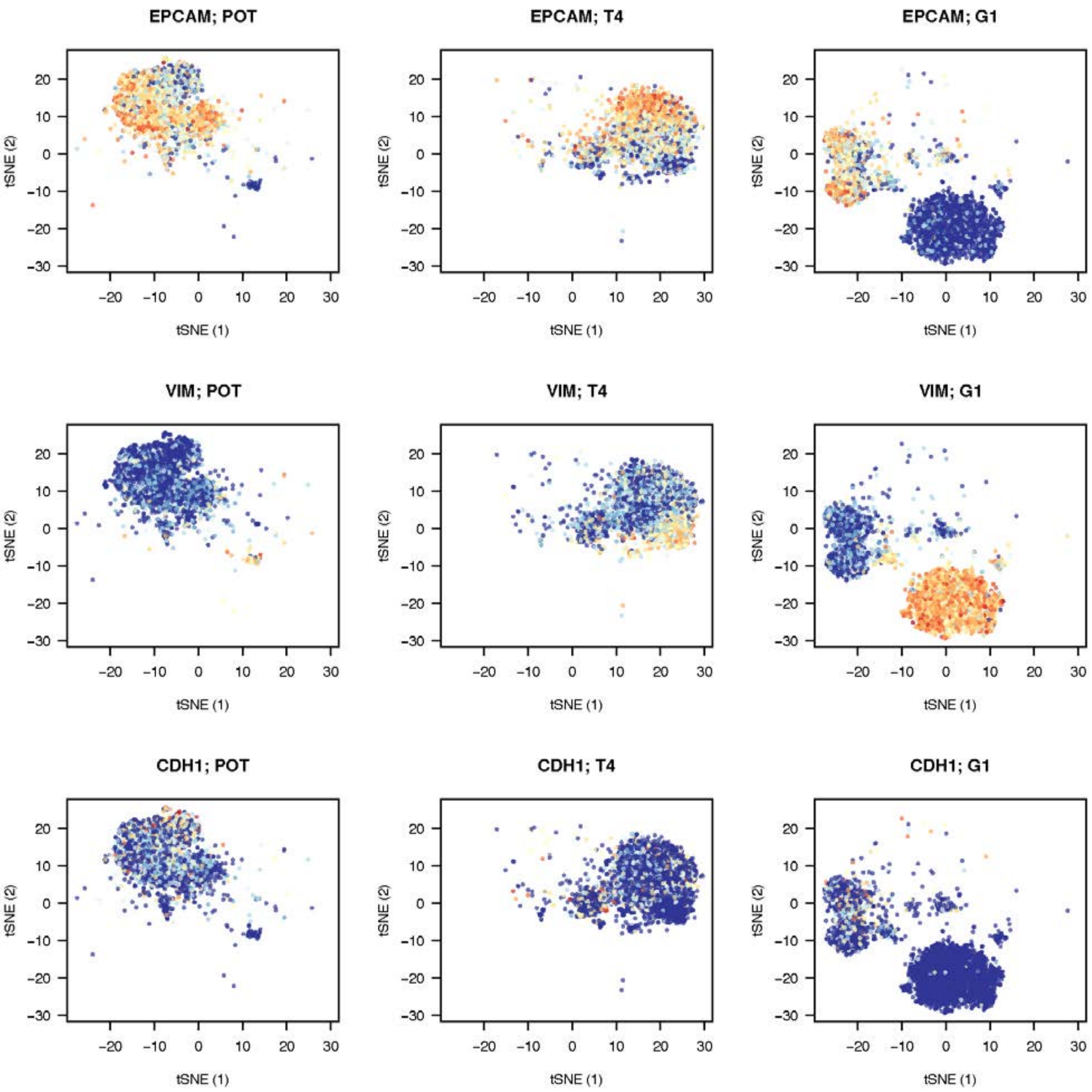

Supplementary Figure 12. Per-gene expression in the single-cell analysis. Expression of genes of interest is reported in the tSNE plots for each sample.

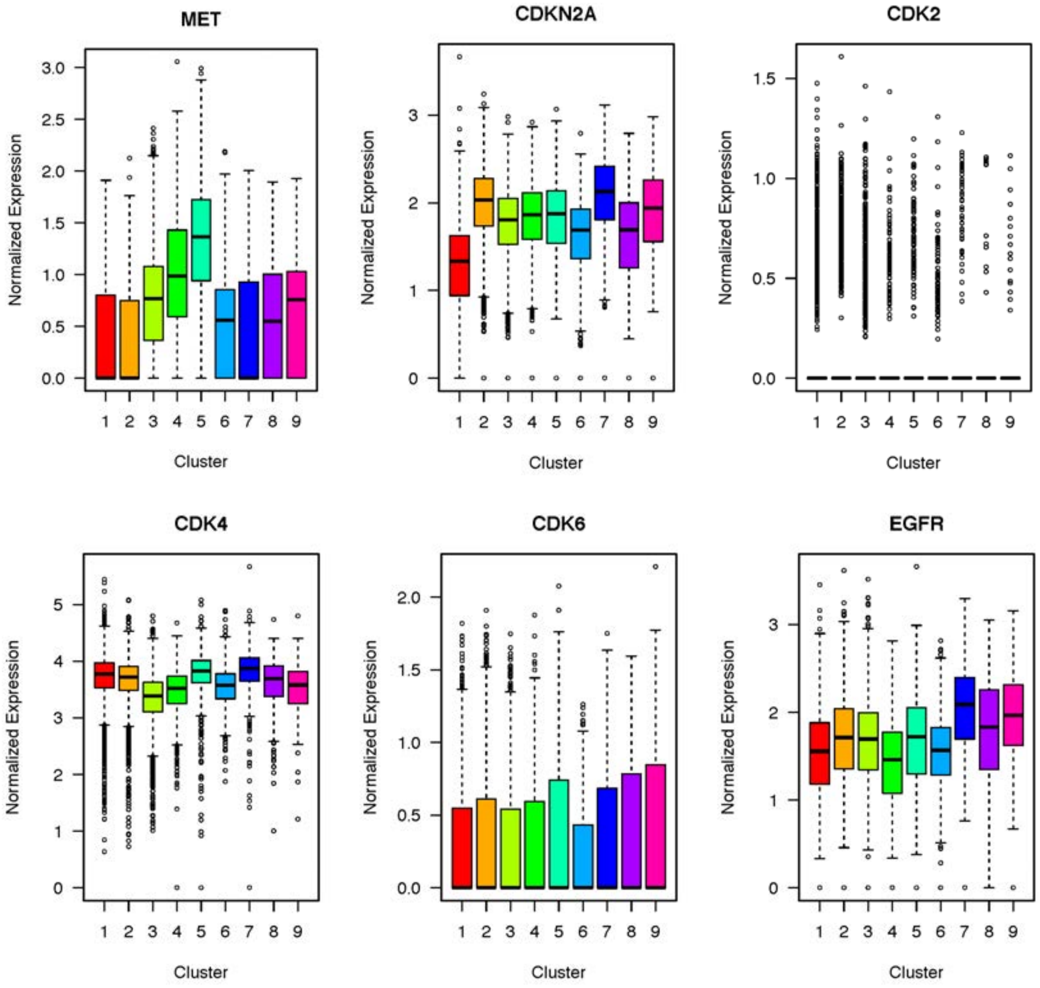
MET
CDKN2A
CDK2
CDK4
CDK6
EGFR
Normalized Expression
Cluster
1 2 3 4 5 6 7 8 9

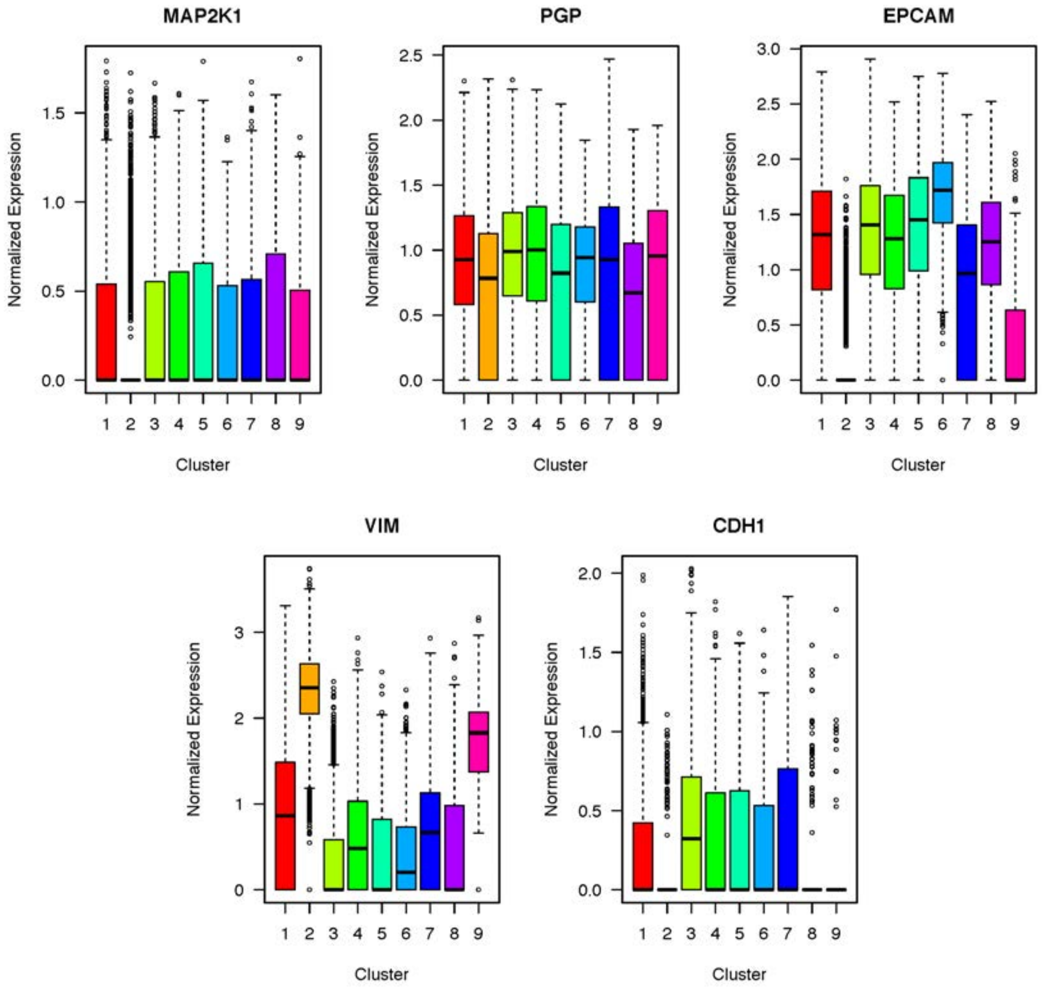

Supplementary Figure 13. Normalised expression of genes of interest per tSNE cluster. For each tSNE cluster from Figure 5B we report the normalised expression of a set of genes of interest. Box plots show median, interquartile values, range and outliers (individual points).

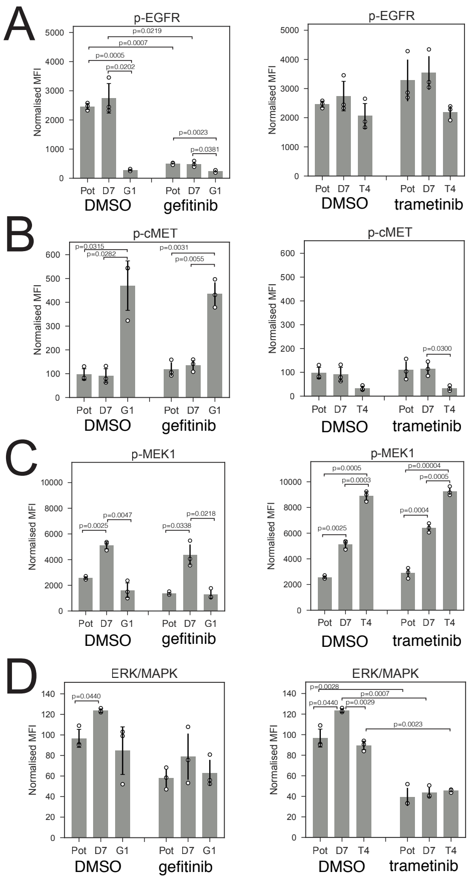

Supplementary Figure 14. Phosphoproteomics confirms drug action on signalling pathways. (A) *EGFR* phosphorylation was highly downregulated under gefitinib and even in the absence of drug in GEF evolved lines, suggesting a stable phenotype where *EGFR* signalling has been lost due to clonal evolution. **(B)** MET phosphorylation was upregulated only in *MET* amplified GEF lines, as expected. **(C)** MEK phosphorylation was variable, however we confirmed *ERK/MAPK* downregulation under trametinib **(D)**, an indication that the drug is inhibiting the *MEK* pathway. Data represent mean normalised MFI (n=3) and error bars are determined by standard deviation, p-values are determined via a two-sided Welch's t-test.

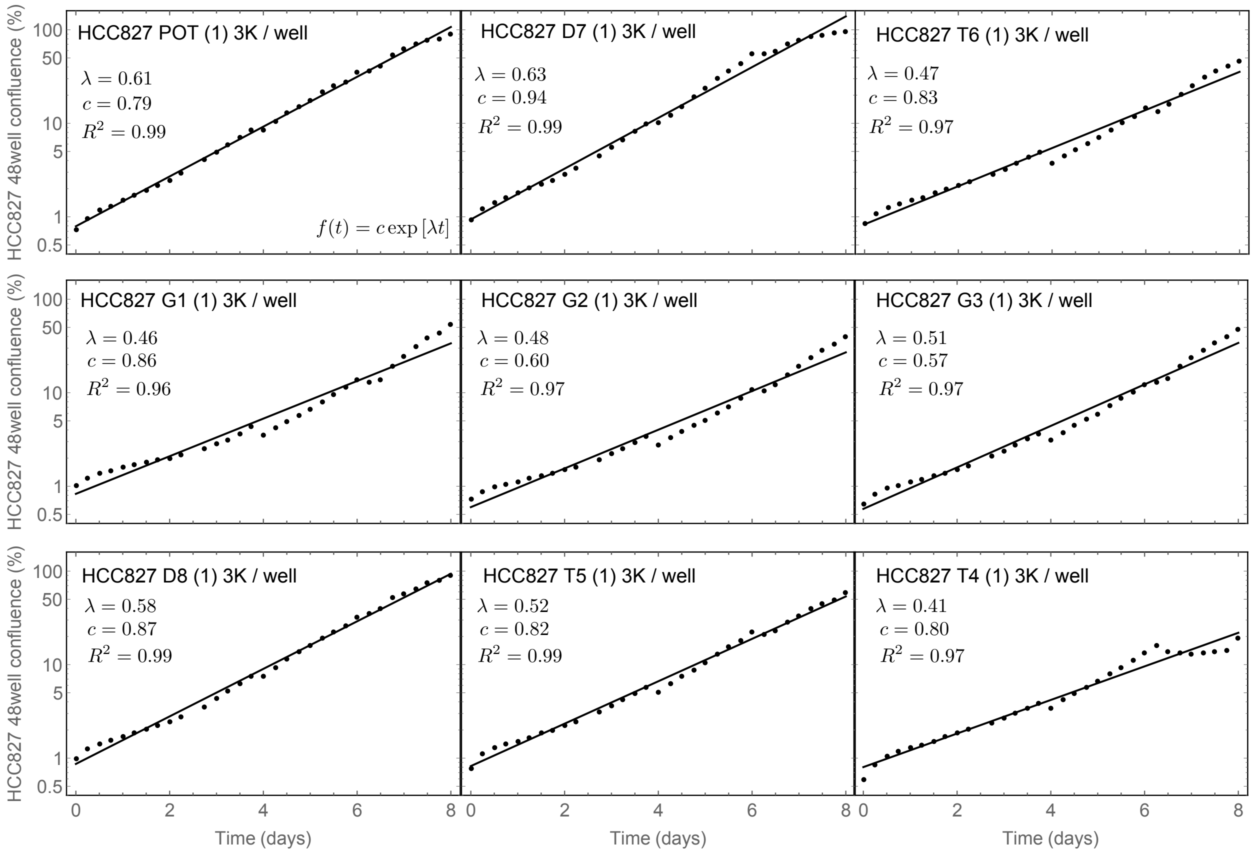

Supplementary Figure 15. Growth rates for each line. We calculated the growth rate of each line observed over time using Incucyte® Live Cell Analysis and linear fitting of log-transformed data. Fit quality was very high, consistent with exponential growth of the lines. Growth rates (lambda) were significantly lower in evolved lines.

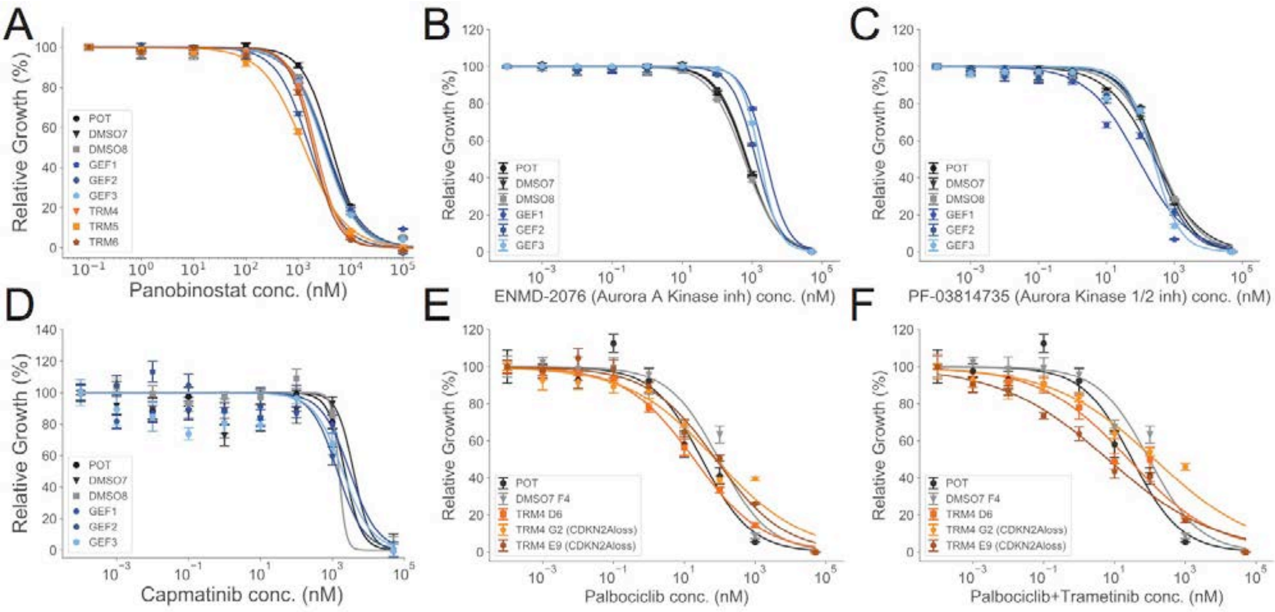

Supplementary Figure 16. Collateral drug sensitivity of additional compounds. We did not find significant collateral drug sensitivity in our evolved lines for panobinostat **(A)**, Aurora A Kinase inhibitors **(B,C)**, capmatinib in bulk samples **(D)** or palbociclib alone **(E,F)**. Error bars represent SEM.

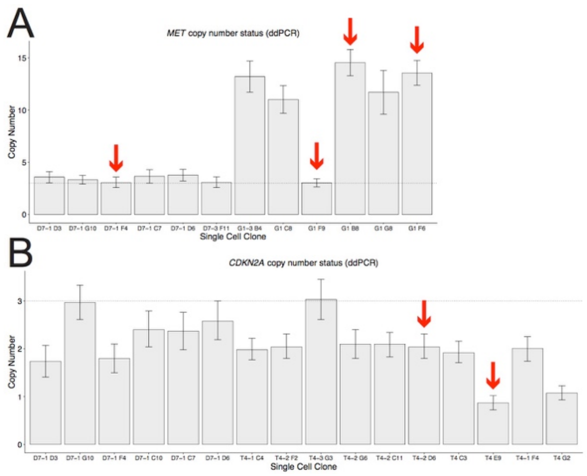

Supplementary Figure 17. ddPCR on single clones. ddPCR for MET **(A)** and CDKN2A **(B)** confirm genomic alteration in a subset of the single clones derived from bulk evolved populations. Bar plots represent the copy number as estimated by dividing the target locus concentration by the reference *NSUN3* locus concentration and multiplying this ratio by three as *NSUN3* is in three copies (triploid genome). Error bars represent the 95% Confidence Interval for the ratio (Total Error Model) as produced by QuantaSoft^{TM} multiplied by 3. In ddPCR it is possible to calculate confidence intervals from the results of a single well by modelling positive and negative droplets as being generated by a Poisson distribution.

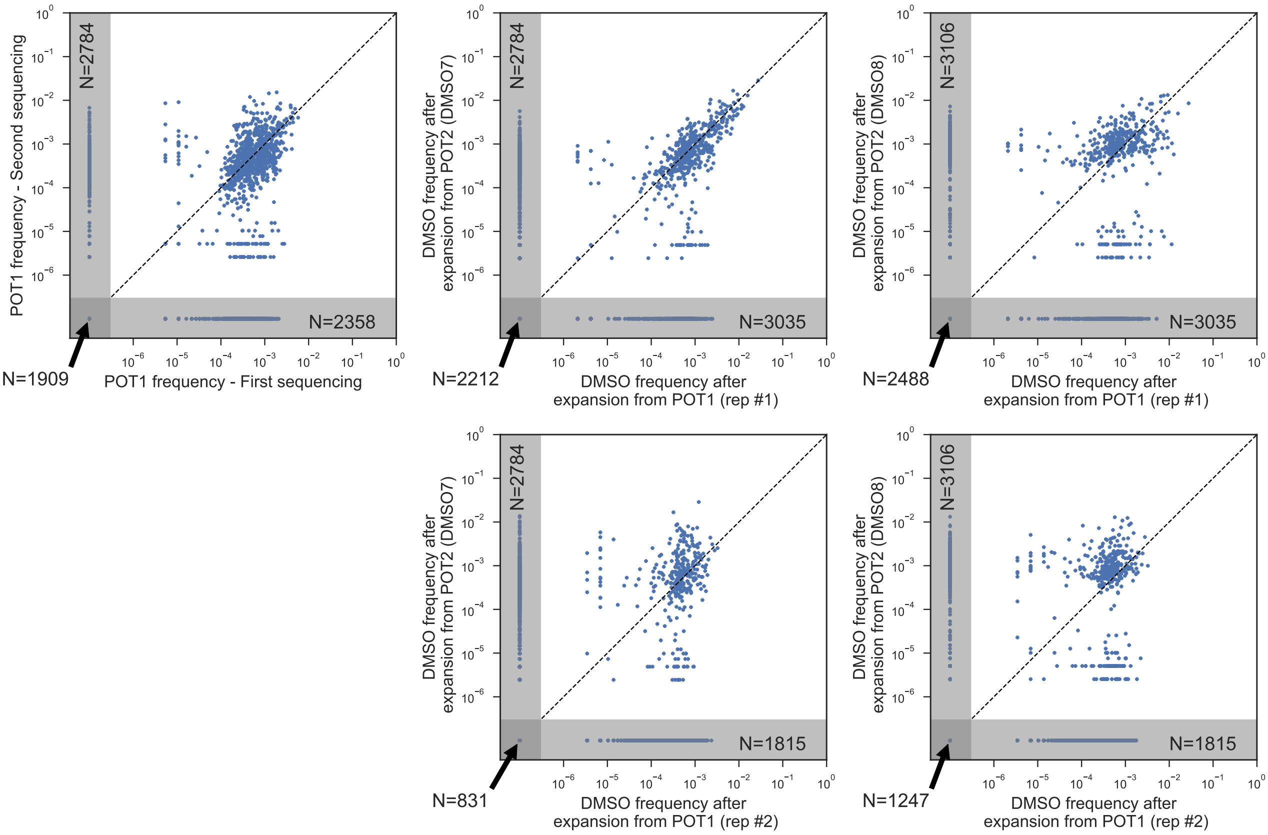

Supplementary Figure 18. Effects of freezing and thawing on barcode frequencies. The frequencies of all barcodes identified in DMSO7 and DMSO8 are consistent to the frequencies in replicates expanded from the POT population before it was frozen. Some barcodes are always missed due to sequencing (binomial sampling of alleles).

Supplementary Methods

Statistical Analysis of Lentiviral Barcoding

During the barcoding protocol, cells are randomly infected with barcodes such that one cell may receive multiple barcodes and that one barcode may appear in multiple cells. Following the statistical approach outlined by Lan et al. [3], we estimated the expected proportion of doubly barcoded cells and the expected proportion of barcodes that appear in multiple cells.

A brief overview of the approach of Lan et al. [3] proceeds as follows. Denote by N_b the total number of barcodes, p_b the probability of a barcoding event, and n_b the number of barcodes received by a single cell. Then $n_b \sim \text{Poisson}(\nu)$ where $\nu = N_b p_b$. Thus,

$$\begin{aligned} P_0 &= \mathbb{P}(n_b = 0) = e^{-\nu} \\ P_1 &= \mathbb{P}(n_b = 1) = \nu e^{-\nu} \\ P_{>1} &= \mathbb{P}(n_b > 1) = 1 - (1+\nu)e^{-\nu}. \end{aligned}$$

ν can be estimated from the barcoding efficiency, η, defined as a the proportion of cells that are successfully labelled with at least one barcode. Specifically $\nu = -\log(1-\eta)$.

We estimate $\eta = 0.1$ as the proportion of cells which survive following selection with puromycin. Thus,

$$\begin{aligned} P_0 &= 0.9 \\ P_1 &= 0.095 \\ P_{>1} &= 0.005. \end{aligned}$$

Now, denote by N_c the total number of cells prepared for lentiviral barcoding, and let n_c be the number of cells receiving a specific barcode. Then $n_c \sim \text{Poisson}(\kappa)$ where $\kappa = p_b N_c = \frac{\nu N_c}{N_b}$. Thus,

$$\begin{aligned} R_0 &= \mathbb{P}(n_c = 0) = e^{-\kappa} \\ R_1 &= \mathbb{P}(n_c = 1) = \nu e^{-\kappa} \\ R_{>1} &= \mathbb{P}(n_c > 1) = 1 - (1+\kappa)e^{-\kappa}. \end{aligned}$$

Hyo-eun et al. [2] report a total barcode library complexity $N_b = 7.2 \times 10^7$ by fitting a polynomial

equation. The total number of cells prepared for barcoding was $N_c = 10^7$. Thus,

$$R_0 = 0.986$$
$$R_1 = 0.014$$
$$R_{>1} < 0.001.$$

Finally, the proportion of uniquely labelled cells (those that receive a unique combination of one or more barcodes) is given by

$$Q \approx \frac{1 - P_0^{R_0}}{1 - P_0^{-1}} = 0.985.$$

Barcode Sequence Merging

Barcodes from Harvested Cells

Errors introduced during PCR or sequencing of the molecular barcodes can result in spurious barcodes being identified, or in the underestimation of the prevalence of a specific barcode. We implemented a novel, bias free error correction protocol as follows.

All reads matching the 12bp of the forward primer, followed by a 30bp sequence, followed by 12bp of the reverse primers were considered. This permits us to identify barcodes that deviate from the weak/strong base pair pattern as a result of errors. Reads were filtered for base quality score >20 in all positions. All detected barcodes were merged into a single file to ensure that the same corrections were applied between different samples.

We next constructed a graph in which the vertices are unique barcodes and the edges connect barcodes which differ by Hamming distance at most 2. We propose that the connected components of this graph represent groups of barcode sequences derived from the same true barcode, and thus that connected components (CCs) should be merged into single representative barcodes. A total of 4777 connected components were extracted. Connected components not containing a representative barcode matching the weak/strong barcode pattern were discarded (60 CCs containing 103 unique barcodes and 12914 total post-filtering reads, 0.0032% of all of the post–filtering reads).

A representative barcode was selected from each CC as the most abundant barcode matching the the weak/strong pattern. Where this representative was >10× more abundant than every other barcode in the CC, all others were corrected to the representative. Where there existed additional barcodes matching the weak/strong pattern and >0.1× the representative count, we designated these barcodes as alternative representatives. 46 CCs were identified as containing multiple representatives (ranging from 2-5 representatives). For these CCs the barcodes were split amongst the representatives by correcting each barcode to the representative with the smallest

Hamming distance. Where multiple representatives had the same Hamming distance, the count for the barcode was evenly split between the representatives to avoid bias.

Barcodes from Supernatent Cells

For the barcodes extracted weekly from the supernatent cells, the extraction and filtering were performed as above. The correction mapping derived from harvested cells was used to correct the barcodes. There were no barcodes identified in the supernatent cells that was not detected in at least one of the harvested samples.

To demonstrate that each of the 8 HYPERflask replicates harbours a suitably similar barcode distribution following preparation, we performed a stochastic population simulation of the POT outgrowth and splitting steps to estimate the likelihood that a barcode in an initial population is present in $N/8$ of the replicate populations. The simulation comprised two parts:

1. Stochastic simulation of the POT outgrowth from an initial population of uniquely barcoded cells.

2. Stochastic simulation of splitting the POT population into 8 replicate populations.

To achieve (1) we assumed that each cell in the initial population was uniquely barcoded, and that each uniquely barcoded population was governed by stochastic exponential growth with birth rate b and death rate d. We implemented a Gillespie algorithm to simulate the exponential growth (Supplementary Figure 2), see Erban et al. [1] for implementation details. Approximate birth and death rates for the HCC827 cell line were previously derived by Mumenthaler et al. [4]. For the oxygen concentration of 20% and media glucose concentration of 2g/L that correspond to our experimental design, the appropriate values are approximately $b = 0.032$, $d = 0.002$, which we used to parameterise the model. Figure 2(B) shows a histogram of population sizes from 10,000 realisations of the simulation from a single cell with instances of extinction (population size equals zero) omitted.

Under this model of stochastic exponential growth differently barcoded populations do not interact, and so the POT barcode frequency distribution was computed by combining 10,000 independent realisations of the stochastic process. The barcode frequency distribution that arises is shown in Figure 2(C).

Finally, to simulate step (2) we performed a random equal size 8 way split of the full population of barcodes generated by the stochastic simulation. To determine the likelihood that a barcode appears in precisely N/8 replicates we simulated the stochastic outgrowth of the POT 10 times, each with 20 associated stochastic simulations of the split, and averaged the results. The predictions are shown in Figure 2(D). We find that 90% of the barcodes that survive the

POT outgrowth appear in 8/8 replicates, with an additional 4% and 2% appearing in 7/8 and 6/8 respectively. Approximately 0.01% of barcodes appear in precisely one replicate.

References

1. Radek Erban, Jonathan Chapman, and Philip Maini. A practical guide to stochastic simulations of reaction-diffusion processes. *arXiv preprint arXiv:0704.1908*, 2007.

2. C Bhang Hyo-eun, David A Ruddy, Viveksagar Krishnamurthy Radhakrishna, Justina X Caushi, Rui Zhao, Matthew M Hims, Angad P Singh, Iris Kao, Daniel Rakiec, Pamela Shaw, et al. Studying clonal dynamics in response to cancer therapy using high-complexity barcoding. *Nature medicine*, 21(5):440, 2015.

3. Xiaoyang Lan, David J Jörg, Florence MG Cavalli, Laura M Richards, Long V Nguyen, Robert J Vanner, Paul Guilhamon, Lilian Lee, Michelle M Kushida, Davide Pellacani, et al. Fate mapping of human glioblastoma reveals an invariant stem cell hierarchy. *Nature*, 549 (7671):227, 2017.

4. Shannon M Mumenthaler, Jasmine Foo, Nathan C Choi, Nicholas Heise, Kevin Leder, David B Agus, William Pao, Franziska Michor, and Parag Mallick. The impact of microenvironmental heterogeneity on the evolution of drug resistance in cancer cells. *Cancer informatics*, 14:CIN–S19338, 2015.
