# Supplementary material for: Exploiting evolutionary steering to induce collateral drug sensitivity in cancer
Source: Nat Commun. 2020 Apr 21;11:1923. doi: 10.1038/s41467-020-15596-z (PMC7174377; doi:10.1038/s41467-020-15596-z)
Supplement: Supplementary file 3 — Description of Additional Supplementary Files [file 41467_2020_15596_MOESM3_ESM.pdf]

Description of Additional Supplementary Files

File Name: Supplementary Data 1
Description: Copy number estimates from whole-exome sequencing.

File Name: Supplementary Data 2
Description: Barcode frequencies.

File Name: Supplementary Data 3
Description: Coverage values of whole-exome sequencing per sample.

File Name: Supplementary Data 4
Description: Single nucleotide variant calls.

File Name: Supplementary Data 5
Description: List of compounds tested in the high-throughput drug screening.
